# Deciphering Resistome and Virulome Diversity in a Porcine Slaughterhouse and Pork Products Through Its Production Chain

**DOI:** 10.3389/fmicb.2018.02099

**Published:** 2018-09-12

**Authors:** Guillermo Campos Calero, Natacha Caballero Gómez, Nabil Benomar, Beatriz Pérez Montoro, Charles W. Knapp, Antonio Gálvez, Hikmate Abriouel

**Affiliations:** ^1^Área de Microbiología, Departamento de Ciencias de la Salud, Facultad de Ciencias Experimentales, Universidad de Jaén, Jaén, Spain; ^2^Centre for Water, Environment, Sustainability & Public Health, Department of Civil and Environmental Engineering, University of Strathclyde, Glasgow, United Kingdom

**Keywords:** antibiotic resistance, slaughterhouse, resistome, virulome, bacteria

## Abstract

We aimed to better understand resistome and virulome patterns on animal and process-area surfaces through a pig slaughterhouse to track possible contamination within the food production chain. Culture-dependent methods revealed high levels of microbial contamination, corresponding to mesophilic and pathogenic bacteria on both the animal and process-area surfaces mainly in the anesthesia (AA and AS) zone followed by “scorching and whip” (FA and FS) zone and also in the end products. To evaluate the potential risk of antibiotic resistance and virulence determinants, shotgun metagenomic DNA-sequencing of isolates from selected areas/products uncovered a high diversity and richness of antibiotic resistance genes (ARGs): 55–62 genes in the anesthesia area (AA and AS) and 35–40 in “animal-arrival zone” (MA and MS). The “scorching and whip” (FA and FS) area, however, exhibited lowered abundance of ARGs (1–6), indicating that the scalding and depilating process (an intermediate zone between “anesthesia” and “scorching and whip”) significantly decreased bacterial load by 1–3 log_10_ but also diminished the resistome. The high prevalence of antibiotic-inactivating enzyme genes in the “animal-arrival zone” (60–65%) and “anesthesia” area (56%) were mainly represented by those for aminoglycoside (46–51%) and lincosamide (14–19%) resistance, which did not reflect selective pressures by antibiotics most commonly used in pig therapy—tetracyclines and beta-lactams. Contrary to ARGs, greater number of virulence resistance genes were detected after evisceration in some products such as kidney, which reflected the poor hygienic practices. More than 19 general virulence features—mainly adherence, secretion system, chemotaxis and motility, invasion and motility were detected in some products. However, immune evasion determinants were detected in almost all samples analyzed from the beginning of the process, with highest amounts found from the anesthesia area. We conclude that there are two main sources of contamination in a pig slaughterhouse: the microorganisms carried on the animals’ hide, and those from the evisceration step. As such, focussing control measures, e.g., enhanced disinfection procedures, on these contamination-source areas may reduce risks to food safety and consumer health, since the antibiotic and virulence determinants may spread to end products and the environment; further, ARG and virulence traits can exacerbate pathogen treatments.

## Introduction

Antibiotic resistance is a natural phenomenon, ancient, highly diverse and globally distributed (Hecker et al., 2003; Davies and Davies, 2010; Pawlowski et al., 2016), which has become elevated during the antibiotic era. Consequently, the selective pressure exerted by antibiotics has dramatically increased drug-resistant bacteria (pathogens and commensals) (Fair and Tor, 2014). The use, misuse and abuse of antibiotics in veterinary, agriculture and clinical therapy for decades have increased the prevalence of resistance genes (ARGs), especially the acquired resistance elements by horizontal gene transfer into the human and animal microbiomes (Laxminarayan, 2014). Additionally, these resistance determinants present on mobile genetic elements have increased the risk of their transfer between different ecosystems. Despite the increasing concerns over inappropriate use of antibiotics in veterinary medicine and food production, slaughterhouse and meat products remain potential reservoirs of antimicrobial resistant bacteria (ARB) and antimicrobial resistance genes (Lavilla Lerma et al., 2013, 2014a,b; Zhu et al., 2013). Consequently, ARB and ARGs can spread to humans throughout the food-supply chain (e.g., Antibiotic Resistance from the Farm to the Table, 2014) by exposure via contaminated animals, meat products, or natural environment (i.e., air, water, and soil) (Founou et al., 2016).

As such, the slaughterhouse environment poses as a potential source and dissemination route for ARGs and ARB contamination. Several steps in the slaughterhouse production system could play crucial roles in the transmission of antimicrobial resistance (AMR) to humans via environmental interfaces and meat products. Preslaughter conditions (e.g., feeding and stabling) can become contaminated via skin and feces, and adherence features in bacteria such as attachment and their biofilm formation capacity can enhance the cross-contamination potential (Koo et al., 2013). Thus, contamination with antibiotic resistant bacteria may occur, increasing the risk of antibiotic resistance gene spread during subsequent slaughtering processes and end products. Within the slaughterhouse, bleeding, evisceration and other related processes can contaminate carcasses and equipment, leading to the spread of gut bacteria (Lavilla Lerma et al., 2013). The gut microbiome often becomes particularly problematic since they represent a complex ecosystem and a epicenter of horizontally transferrable resistance traits between commensals and pathogens (Carlet, 2012), and as such, they cross-transmit resistant strains between animals and humans (Leverstein-van Hall et al., 2011; Overdevest et al., 2011; Economou and Gousia, 2015). On the other hand, the transfer of antibiotic resistance from human to animals may also occur due to the some interconnection process between ecosystems. Further, many commonly used biocides for disinfection procedures (Chuanchuen et al., 2001; Gilbert et al., 2002) promote cross-resistance with antibiotics due to their action on common targets (Lavilla Lerma et al., 2014a,b); thereby shifting bacteria from having a single resistance trait leading to the selection of multidrug- or pan-drug resistant bacteria (Magiorakos et al., 2012).

Advanced molecular technologies, such as metagenomic sequencing to detect and quantify resistome-virulome of an entire microbial population (Baquero et al., 2013), allow us to predict, prevent and manage antibiotic resistance and virulence determinants in several environments (Noyes et al., 2017). This remains an excellent methodological approach for determining types and abundances of ARGs and virulence elements, which can further provide information about the cumulative proliferation of antibiotic resistance within a system (Luby et al., 2016). In this study, shotgun metagenomic sequencing of cultured isolates from different process-area and animal surfaces, and the resulting meat products, was conducted to determine the microbial and the resistome-virulome diversity thorughout a pig slaugtherhouse in Jaén (Spain). It remains crucial to understand and localize the focus of resistant and virulent bacteria in a slaughterhouse to reduced their spread and impact on the environment.

## Materials and Methods

### Sample Collection and Processing

**FIGURE 1:**
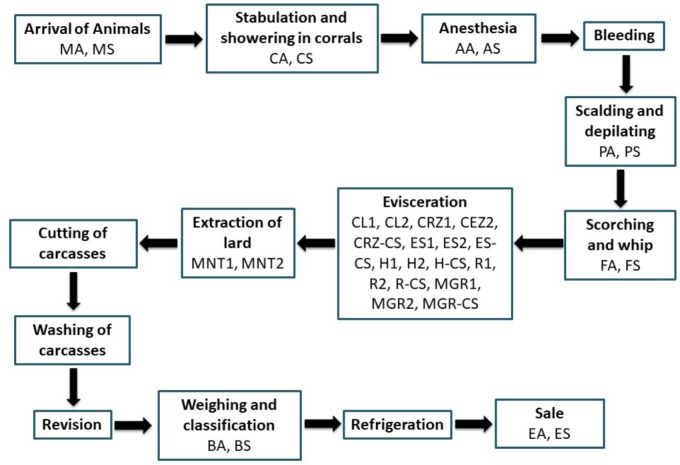
Flowchart of meat chain production in a pig slaughterhouse of Jaeìn. Samples were taken from animal (A) and also environmental (S) surfaces. CL1 and CL2, multiple transport box samples; CRZ1 and CRZ2, heart surface samples; CRZ-CS, heart box sample; ES1 and ES2, stomach surface samples; ES-CS, stomach box sample; H1 and H2, liver surface samples; H-CS, liver box sample; R1 and R2, kidney surface samples; R-CS, kidney box sample; MGR1 and MGR2, lean surface samples; MGR-CS, lean box sample.

This study involved sample isolates collected from a local pig slaughterhouse (Jaén, Spain), which is representative of those in the region and receives animals from multiple suppliers and geographic locations. Different samples were collected with sterile swabs from the animals’ backs (A; or equivalent surfaces) and also environmental surfaces (S) from the following zones (**Figure 1**): “animal arrival” (MA, MS), stabling and corral showers (CA, CS), anesthesia (AA, AS), “scalding and depilating” (PA, PS), “scorching and whip” (FA, FS), evisceration (several samples) and extraction of lard (MNT1, MNT2), “weight and classification” (BA, BS) and sale (EA, ES). For animal products, only the surface was swabbed. The samples were immediately stored and transported to the laboratory under refrigerated conditions.

### Microbiological Analyses of Culturable Microbiota

The presence of various groups of bacteria throughout meat production chain were determined from animal and slaughterhouse surfaces: total aerobic mesophilic bacteria, *Staphylococcus aureus*, *Listeria monocytogenes*, *Escherichia coli*, *Salmonella* sp., pseudomonads and lactic acid bacteria (LAB) (Lavilla Lerma et al., 2013). Sterile swabs were used to collect samples (100 cm^2^) from the selected sites (described above) being this operation repeated eight times; they were then immersed each one in 1 ml of sterile saline solution (0.85%; Scharlab, Barcelona, Spain) and stored at 4°C for 24 h to release bacteria from swabs while minimizing growth. Then, samples (eight replicates) were pooled to provide a representative microbiota community from each zone, and they were then serially diluted in sterile saline solution and plated in triplicate on the following media: Tryptone Soya Agar “TSA” (Scharlab, Barcelona, Spain) for estimation of total aerobic mesophilic bacteria, Baid Parker Agar “BPA” (Scharlab, Barcelona, Spain) for staphylococci, Palcam Agar “PA” (Scharlab, Barcelona, Spain) for *L. monocytogenes*, TBX (Sigma-Aldrich, Madrid, Spain) for *E. coli*, XLD (Scharlab, Barcelona, Spain) for *Salmonella* sp., King Agar “KA” (Scharlab, Barcelona, Spain) for pseudomonads (King et al., 1954), “MRSA” (Scharlab) supplemented with 0.4 g/l sodium azide (Scharlab, Barcelona, Spain) for LAB (Ahn et al., 2002). Counts from the different inoculated media were obtained after 48 h of incubation at 37°C (TSA, BPA, PA, TBX, XLD) and after 72 h of incubation at 30°C (MRSA) and 22°C (KA). Results were calculated as the mean of three determinations.

### Resistome Determination in Pig Slaughterhouse Throughout Meat Production Chain

#### Total DNA Extraction

The pooled saline samples, as previously mentioned, were used for DNA community analysis. The following animal/environmental sampls were selected for their microbial contamination and because they represented slaughterhouse sufficient processes to gain an image of antibiotic resistance spread: “animal arrival” (MA and MS), anesthesia (AA from and AS), “scorching and whip” (*chamuscado y flagelado*, FA and FS), evisceration process (kidney R1 and R2; lean MGR1 and MGR2), and sale (EA and ES) zones. Bacterial samples, following 24 h incubation at 4°C, were centrifuged (14000 rpm for 10 min), and the DNA were extracted using ZymoBIOMICS DNA Miniprep Kit (Zymo Research, California, United States) according to the manufacturer’s instructions. Total DNA quantification and quality were assessed by a NanoDrop 2000 spectrophotometer (Thermo Scientific). DNA samples were frozen at -80°C until required.

#### Construction of Metagenomic Libraries

Construction of the metagenomic libraries was done using the Illumina Nextera XT DNA Library Prep Kit (Illumina, Inc., San Diego, CA, United States) according to the manufacturer’s instructions. The resulting DNA was pooled by equivalent weight. Library sequencing was done in NextSeq 500 platform (2 × 150 bp read lengths) at Lifesequencing S.L. (Valencia, Spain). The resulting reads were assembled using SPAdes program version 3.10. Assembly and annotation were done at Lifesequencing S.L. (Valencia, Spain). The mean coverage for the kmer 99 ranged between 1.68 and 6.35.

#### Antibiotic Resistance Gene Detection

The predicted CDSs (coding DNA sequences) were annotated using BLAST (Basic Local Alignment Search Tool) against the CARD’s (Comprehensive Antibiotic Resistance Database) curated AMR (antimicrobial resistance) database with the aim to detect antibiotic resistance genes (ARG) using Resistance Gene Identifier RGI v3.2.1 (as part of CARD tools). Furthermore, the RGI software (RGI 3.2.1) used for prediction of resistome from protein or nucleotide data has been based on homology and SNP (Single Nucleotide Polymorphism) models. Resistome sequences have been deposited at the EMBL Nucleotide Sequence Database under accession numbers ERX2604238 to ERX2604249.

#### Virulence Determinant Detection

The predicted CDSs were annotated by using reciprocal BLAST and VFDB (Virulence Factors of Bacterial Pathogens) database. Obtained hits were considered positive when the results of reciprocal BLAST were similar, with a 80% sequence similarity cut-off. Virulome sequences has been deposited at the EMBL Nucleotide Sequence Database under accession numbers ERX2604238 to ERX2604249.

### Statistical Analyses

All analyses were performed in triplicate. Statistical analyses were conducted using Excel 2007 (Microsoft Corporation, Redmond, WA, United States) program to determine averages and standard deviations. Statistical treatment of data was conducted by analysis of variances (ANOVA) in Statgraphics Centurion XVI software using Shapiro–Wilk test and the Levene test to check data normality and the 2-sided Tukey’s test to determine the significance of differences between strains, where P < 0.05 was considered statistically significant.

## Results

### Microbial Diversity in a Pig Slaughterhouse Throughout Meat Chain Production

Culture dependent-methods revealed many populations from several bacterial groups recovered from animal and environmental surfaces of a pig slaughterhouse and meat processing plant in Jaén (**Figure 1** and **Table 1**). The largest number of mesophilic bacteria was recovered from the first steps of slaughtering like stabling and showers (CA, CS), anesthesia (AA, AS), and “scorching and whip” (FA, FS) and approached 7.71 log_10_ CFU/ml (**Table 1**). No differences were detected between animal and environmental/processing surfaces (counts ranging from 4.41–7.71 log_10_ CFU/ml); however, no bacteria were recovered from the final step of meat production before shipment, i.e., the surface within the sale zone (ES). The counts of pseudomonads recovered from animal and process-area surfaces were similar, ranging from 3.84 to 7.48 log_10_ CFU/ml with the lowest counts in “weights and classification” (BA, BS) and sale (EA, ES) zones (**Table 1**). Staphylococci were also detected in all samples (animal and environment) ranging from 3.21 to 7.15 log_10_ CFU/ml and, in a similar manner, the lowest counts were registered in “weight and classification” (BA, BS) and sale (EA, ES) zones (**Table 1**). Regarding *L. monocytogenes*, this pathogen was highly recovered from animal surface in anesthesia (AA) zone and process surfaces in “scorching and whip” (FS) reaching 5.77 and 6.30 log_10_ CFU/ml, respectively (**Table 1**). However, the lowest counts were registered in animal surface from “animal-arrival zone” (MA) (0 log_10_ CFU/ml), surfaces and animal products in the sale zone (ES and EA: 0 log_10_ CFU/ml and 1 log_10_ CFU/ml, respectively) (**Table 1**). On the other hand, *E. coli* was detected in all animal and process-area surfaces except on animal surfaces following “scalding and depilating” (PA) and on the surfaces in the sale zone (ES: 0 log_10_ CFU/ml) (**Table 1**). Indeed, lower *E. coli* populations were detected on surfaces of animal products in the sale zone (EA) and environmental surfaces in “weight and classification” (BS) of 1 and 1.15 log_10_ CFU/ml, respectively (**Table 1**). However, highest counts were obtained on animal and process-area surfaces in the anesthesia zone (AA and AS) at 5.79–5.91 log_10_ CFU/ml (**Table 1**). Concerning *Salmonella* sp., animal and environmental surfaces in anesthesia zone (AA and AS) showed the most contaminated surfaces in slaughterhouse being 4.20–5.30 log_10_ CFU/ml (**Table 1**). However, *Salmonella* sp. was absent in animal surface of Arrival of Animals zone (MA), animal surface of “scalding and depilating” zone (PA), animal and environmental surfaces of “scorching and whip” (FA and FS) and sale (EA and ES) zones (**Table 1**). Lactic acid bacteria were destected in all zones especially on animals in anesthesia and process-area surfaces (AA and AS) and animal surfaces in “stabling and corral showers” (CA) ranging from 6.64 to 6.8 log_10_ CFU/ml (**Table 1**). However, the other zones showed intermediate counts ranging from 3.4 to 5.01 log_10_ CFU/ml (**Table 1**).

**Table 1:** Microbial counts in animal and environmental surfaces throughout meat chain production in a porcine slaughterhouse.

Culture media	Microbial counts in animal and environmental surfaces (CFU/ml)^∗^						
	MA	MS	CA	CS	AA	AS	PA
TSA	5,26 ± 0.07^d^	5.46 ± 0.17^d^	7.05 ± 0.08^f^	5.88 ± 0.01^e^	7.72 ± 0.24^g^	7.55 ± 0.38^g^	5.87 ± 0.06^e^
KA	5.06 ± 0.64^b^	6.54 ± 0.13^ef^	6.96 ± 0.01^fg^	7.02 ± 0.36^fg^	7.11 ± 0.52^h^	7.49 ± 0.21^h^	6.25 ± 0.10^de^
BPA	5.24 ± 0.10^cd^	6.16 ± 0.06^e^	7.12 ± 0.06^f^	6.93 ± 0.06^f^	7.16 ± 0.58^f^	7.02 ± 0.69^f^	5.19 ± 0.07^cd^
PA	0^a^	2.74 ± 0.06^bc^	3.54 ± 0.09^bcd^	3.30 ± 0.43^bcd^	5.78 ± 0.43^de^	3.06 ± 0.43^bc^	2.75 ± 0.21^bc^
TBX	4.38 ± 0.08^cd^	4.98 ± 0.26^de^	4.85 ± 0.00^cde^	4.74 ± 0.06^cde^	5.79 ± 0.05^e^	5.92 ± 0.44^e^	0^a^
XLD	0^a^	1.50 ± 0.21^ab^	3.50 ± 0.28^cde^	2.48 ± 0.00^bcd^	5.30 ± 0.00^f^	4.21 ± 0.13^de^	0^a^
MRSA	4.71 ± 0.00^fg^	4.78 ± 0.34^g^	6.39 ± 0.04^h^	5.01 ± 0.00^g^	6.64 ± 0.21^hi^	6.81 ± 0.14^i^	3.55 ± 0.05^ab^

**Culture media**	**Microbial counts in animal and environmental surfaces (CFU/ml)^∗^**						
	**FA**	**FS**	**BA**	**BS**	**EA**	**ES**	**PS**

TSA	7.61 ± 0.05^g^	7.08 ± 0.18^f^	4.87 ± 0.03^c^	4.45 ± 0.18^b^	4.42 ± 0.16^b^	0^a^	5.90 ± 0.05^e^
KA	5.71 ± 0.07^cd^	7.32 ± 0.09^h^	5.19 ± 0.06^bc^	4.90 ± 0.01^b^	4.72 ± 0.01^b^	3.85 ± 0.01^a^	6.16 ± 0.29^de^
BPA	5.07 ± 0.10^bc^	5.74 ± 0.07^de^	4.53 ± 0.05^b^	3.50 ± 0.03^a^	3.72 ± 0.03^a^	3.21 ± 0.24^a^	5.43 ± 0.28^cd^
PA	4.00 ± 0.06^cde^	6.30 ± 0.00^e^	2.35 ± 0.49^abc^	2.50 ± 0.28^abc^	1.00 ± 0.14^ab^	0^a^	3.66 ± 0.04^cde^
TBX	4.64 ± 0.12^cde^	3.68 ± 0.14^bc^	3.06 ± 0.03^b^	1.15 ± 0.16^a^	1.00 ± 0.14^a^	0^a^	3.98 ± 0.39^bcd^
XLD	0^a^	0^a^	1.35 ± 0.19^ab^	2.24 ± 0.34^bc^	0^a^	0^a^	1.00 ± 0.14^ab^
MRSA	4.28 ± 0.11^de^	4.45 ± 0.15^ef^	3.89 ± 0.02^c^	3.86 ± 0.17^bc^	3.37 ± 0.16^a^	3.41 ± 0.12^a^	4.04 ± 0.11^cd^


*±SD, standard deviations of three independent experiments. ^∗^Each different lower-case letter represents significant differences according to 2-sided Tukey’s HSD between strains (p < 0.05). AA, animal surfaces in Anesthesia zone; AS, process-area surfaces in Anesthesia zone; BA, animal surfaces in weight and classification; BS, process-area surfaces in weight and classification; CA, animal surfaces in stabling and corral showers; CS, process-area surfaces in stabling and corral showers; EA, animal surfaces in Sale zone; ES, process-area surfaces in Sale zone; FA, animal surfaces in Scorching and Whip zone; FS, process-area surfaces in Scorching and Whip zone; MA, animal surfaces in Animal Arrival zone; MS, process-area surfaces in Animal Arrival zone; PA, animal surfaces in Scalding and Depilating zone; PS, process-area surfaces in Scalding and Depilating zone*.

Animal products (stomach, kidney, heart, lean, and lard) obtained after evisceration and also their corresponding transport boxes (H-CS, R-CS, CRZ-CS, ES-CS, MGR-CS, CL1, and CL2) were examined for bacterial diversity in a similar manner as for animal and processing surfaces (**Table 2**). In general, samples from all transporting boxes exhibited greater counts of total mesophilic bacteria (ranging from 6.29 to 7.56 log_10_ CFU/ml) than their corresponding products (ranging from 4.41 to 6.81 log_10_ CFU/ml) (**Table 2**). Similar results were obtained for pseudomonads (ranging from 4.56 to 7.55 log_10_ CFU/ml) (**Table 2**) and staphylococci (ranging from 3.25 to 6.42 log_10_ CFU/ml) (**Table 2**). However, *L. monocytogenes* were only detected in sample R1 from kidney (3.3 log_10_ CFU/ml), sample MGR1 from lean meat (3.02 log_10_ CFU/ml) and sample MNT1 from lard (2.38 log_10_ CFU/ml) (**Table 2**). On the other hand, *E. coli* were detected in all product samples and also transport boxes (ranging from 2.69 to 4.83 log_10_ CFU/ml) except MNT1 (lard sample) and CL1 (transport box for kidney and liver) (**Table 2**). *Salmonella* sp. were recovered from liver surface (H2) and its transport box (H-CS), the transport box of kidney (R-CS), the stomach (ES1), the transport box of lean (MGR-CS) and the transport box (CL2) for several organs (heart, stomach, and lean) being the counts ranging from 2.23 to 3.65 log_10_ CFU/ml (**Table 2**). Regarding LAB, they were present in all animal products and transport boxes (ranging from 3.41 to 6.17 log_10_ CFU/ml) except the transport box CL1 used for kidney and liver (**Table 2**).

**Table 2:** Microbial counts in pork products through its production chain in a porcine slaughterhouse.

Culture media	Product surface (CFU/ml)^∗^									
	H1	H2	H-CS	R1	R2	R-CS	CRZ1	CRZ2	CRZ-CS
TSA	4.86 ± 0.13^b^	5.31 ± 0.05^c^	6.30 ± 0.09^de^	6.34 ± 0.03^def^	4.41 ± 0.05^a^	6.72 ± 0.04^fg^	6.81 ± 0.06^gh^	6.41 ± 0.05^ef^
KA	7.30 ± 0.00^i^	7.30 ± 0.00^i^	7.30 ± 0.00^i^	6.14 ± 0.34^ef^	4.56 ± 0.04^a^	6.80 ± 0.04^h^	6.24 ± 0.09^efg^	5.72 ± 0.17^d^	7.30 ± 0.00^i^
BPA	3.25 ± 0.07^a^	3.64 ± 0.03^b^	5.48 ± 0.07^i^	4.82 ± 0.21^f^	3.62 ± 0.01^b^	4.98 ± 0.09^fg^	5.34 ± 0.07^i^	5.03 ± 0.35^fhg^	5.48 ± 0.03^i^
PA	0^a^	0^a^	0^bc^	3.30 ± 0.14^d^	0^a^	0^a^	0^a^	0^a^
TBX	3.69 ± 0.12^defg^	3.60 ± 0.01^defg^	3.82 ± 0.01^defg^	4.17 ± 0.07^fghij^	3.24 ± 0.18^cde^	3.97 ± 0.02^efghi^	4.58 ± 0.28^hij^	4.27 ± 0.05^ghij^	2.69 ± 0.30^c^
XLD	0^ab^	2.78 ± 0.25^bcd^	2.45 ± 0.21^bcd^	0^a^	0^a^	2.24 ± 0.34^bcd^	0^a^	0^a^	0^a^
MRSA	4.48 ± 0.03^cdefg^	3.94 ± 0.03^bc^	4.44 ± 0.01^cdefg^	4.42 ± 0.06^cdef^	3.41 ± 0.07^b^	4.78 ± 0.13^defg^	4.08 ± 0.15^bcd^	3.48 ± 0.02^b^	4.30 ± 0.01^cde^

**Culture media**	**Product surface (CFU/ml)^∗^**									
	**ES1**	**ES2**	**ES-CS**	**MGR1**	**MGR2**	**MGR-CS**	**MNT1**	**MNT2**	**CL1**	**CL2**

TSA	6.10 ± 0.05^de^	6.00 ± 0.74^d^	7.00 ± 0.21^gh^	6.17 ± 0.12^de^	6.00 ± 0.14^d^	7.56 ± 0.09^i^	5.04 ± 0.08^bc^	4.86 ± 0.03^b^	6.31 ± 0.05^de^	7.57 ± 0.00^i^
KA	6.04 ± 0.01^e^	5.78 ± 0.06^d^	6.74 ± 0.07^h^	6.25 ± 0.10^fg^	6.43 ± 0.07^g^	7.56 ± 0.04^j^	5.11 ± 0.02^c^	4.84 ± 0.03^b^	6.33 ± 0.10^fg^	7.45 ± 0.02^ij^
BPA	4.29 ± 0.02^e^	3.72 ± 0.18^bc^	4.80 ± 0.03^f^	5.29 ± 0.02^hi^	5.27 ± 0.02^ghi^	6.42 ± 0.02^k^	3.99 ± 0.01^cd^	4.00 ± 0.10^cd^	4.18 ± 0.31^de^	5.94 ± 0.13^j^
PA	0^a^	0^a^	0^a^	3.02 ± 0.25^d^	0^ab^	0^a^	2.39 ± 0.12^cd^	0^a^	0^a^	0^a^
TBX	3.94 ± 0.15^defghi^	4.70 ± 0.02^ij^	3.24 ± 0.41^cde^	3.55 ± 0.08^defg^	3.19 ± 0.06^cd^	3.49 ± 0.26^def^	3.83 ± 0.02^defgh^	0^b^	0^a^	4.84 ± 0.06^j^
XLD	3.48 ± 0.01^d^	0^a^	0^a^	0^abc^	0^abc^	3.35 ± 0.49^cd^	0^abc^	0^a^	0^a^	3.65 ± 0.07^d^
MRSA	4.83 ± 0.15^defg^	5.07 ± 0.09^efg^	3.96 ± 0.04^bc^	5.09 ± 0.03^efg^	5.24 ± 0.09^g^	4.63 ± 0.02^cdefg^	5.12 ± 0.04^fg^	4.40 ± 0.14^cdef^	0^a^	6.18 ± 0.06^h^


*±SD, standard deviations of three independent experiments. ^∗^Each different lower-case letter represents significant differences according to 2-sided Tukey’s HSD between strains (p < 0.05). CL1 and CL2, multiple transport box samples (before organ transport); CRZ1 and CRZ2, heart surface samples; CRZ-CS, heart box sample; ES1 and ES2, stomach surface samples; ES-CS, stomach box sample; H1 and H2, liver surface samples; H-CS, liver box sample; R1 and R2, kidney surface samples; R-CS, kidney box sample; MGR1 and MGR2, lean surface samples; MGR-CS, lean box sample; MNT1 and MNT2, lard samples.*

### Determination of Resistome Throughout Meat Chain Production

**FIGURE 2:**
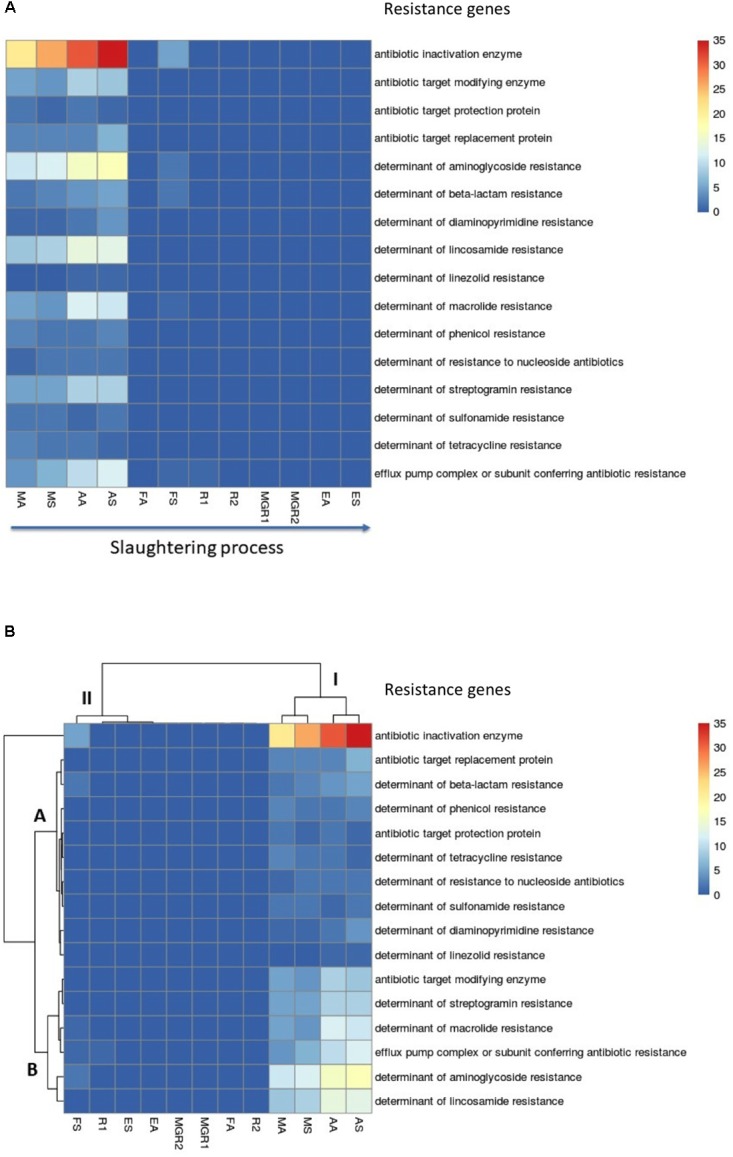
Heatmap showing the distribution of antibiotic resistance genes (ARGs) detected within metagenomic samples of different slaughterhouse zone/product surfaces (MA, animal surfaces in “animal arrival” zone; MS, environmental surfaces in “animal arrival” zone; AA, animal surfaces in anesthesia zone; AS, process-area surfaces in anesthesia zone; FA, animal surfaces in “scorching and whip” zone; FS, process-area surfaces in “scorching and whip” zone; EA, animal surfaces in sale zone; ES, area surfaces in sale zone; R1 and R2, kidney samples; MGR1 and MGR2, lean samples). **(A)** distribution of ARGs throughout meat chain production. **(B)** ARGs clustered by UPGMA (Unweighted Pair Group Method with Arithmetic Mean) method using Euclidean distances showing two mains clusters: cluster I and cluster II. Colors shown in the legend (right) indicated the abundance of ARGs scaled from blue (undetected ARGs) to red (maximum; i.e., 35 ARG).

To assess the resistome throughout the meat production chain in a pig slaughterhouse, we collected pooled samples from animal (or product) surfaces and process surfaces from five selected zones: “animal-arrival” (MA, MS), anesthesia (AA, AS), “scorching and whip” (FA, FS), evisceration (R1 and R2 kidney samples, MGR1 and MGR2 lean samples), and sale (EA, ES) (**Figure 1**). Heatmap (**Figure 2A**) demonstrates ARG abundances found in the surfaces of animals and the environment from the first steps of slaughtering, being almost absent in product surfaces (kidney and lean meat) and also sale zone (**Figure 2A**). Cluster analysis of ARG data showed two main clusters: cluster I, represented by “animal arrival” (MA, MS) and anesthesia (AA, AS) zones, which exhibited high diversity and richness of ARGs up to 62 ARGs (AS), 55 ARGs (AA), 40 ARGs (MS), and 35 ARGs (MA) (**Figure 2B** and **Supplementary Material S1**); however, cluster II was represented by the other zones and animal products [“scorching and whip” (FA, FS), evisceration (R1 and R2) kidney-surface samples, MGR1 and MGR2 lean-surface samples, and sale (EA, ES)] with low abundance of ARGs (**Figure 2B**). The lowest levels of microbiota resistome was observed in kidney surface (R1) and “scorching and whip” process surface (FS) with one ARG and six ARGs, respectively (**Supplementary Material S1**). Furthermore, no ARGs were detected in the rest of zone/product surfaces (EA, ES, FA, MGR1, and MGR2).

Among antibiotic resistance genes, we found 16 different mechanisms of resistance; most abundant were genes encoding for antibiotic-inactivating enzymes with up to 35 genes in AS, and 31 genes in AA, 26 genes in MS, 21 genes in MA and 5 genes in FS (**Figure 2** and **Supplementary Material S1**). Specific genes such as aminoglycoside resistance up to 17 were detected in AS, 16 genes in AA, 12 genes in MS, 11 genes in MA and two genes in FS (**Figure 2A**); and lincosamide resistance, macrolide resistance, efflux-pump complex and streptogramin resistance genes were also notably detected in AS and AA (9–14 genes), MS and MA (4–9 genes). On the other hand, FS only had the presence of one gene coding for macrolide resistance and for efflux-pump complex; however, R1 only showed the presence of one efflux-pump complex coding gene (**Figure 2** and **Supplementary Material S1**).

Cluster analysis of ARGs further revealed that the most abundant determinant type (antibiotic-inactivating enzymes) clustered separately; however, other determinants present in slaughterhouse showed two main clusters: cluster A and cluster B (**Figure 2B**). Cluster A was presented by genes coding for resistance to beta-lactams, phenicols, tetracyclines, nucleoside antibiotics, sulfonamides, diaminopyrimidines and linezolids along with non-specific genes coding for antibiotic-target replacement protein and antibiotic-target protection protein (**Figure 2B**). Cluster B, which was the second most important in resistome cluster following those for antibiotic-inactivating enzymes, was comprised of genes encoding resistance to streptogramin, macrolides, aminoglycosides and lincosamides along with non-specific genes coding for antibiotic-modifying enzymes and efflux-pump complexes (**Figure 2B**).

**FIGURE 3:**
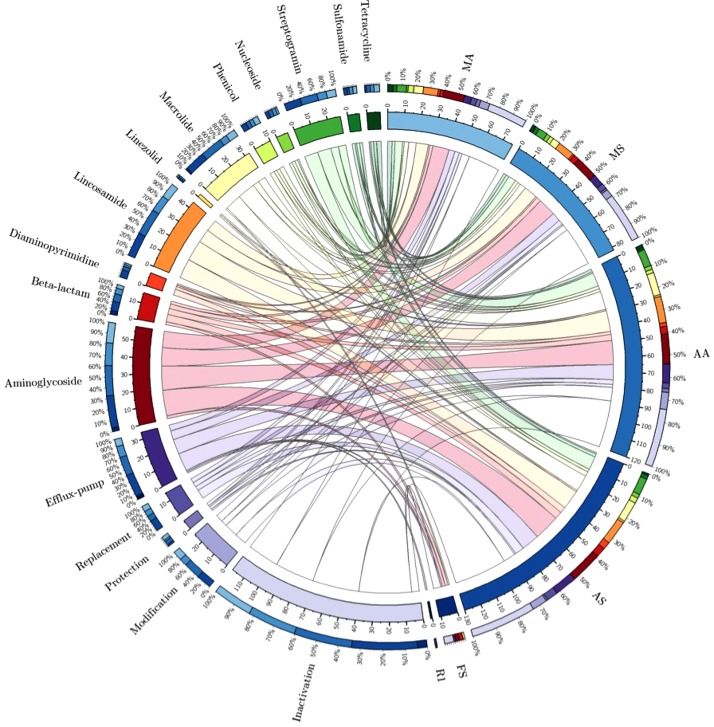
Distributions of antibiotic resistance gene (ARG) types and their abundances in metagenomic results from different slaughterhouse zone/product surfaces (visualized *via* Circos). The length of the bars on the outer-ring represents the percentage of ARGs in each metagenome sample. Each ARG was represented by a specific ribbon color, and the width of each ribbon demonstrates the abundance of each ARG.

On the other hand, the most prevalent antibiotic resistance genes were aminoglycoside (46–51%) and lincosamide (14–19%) resistance genes mainly in animal-arrival zone (MA and MS) and anesthesia area (AA and AS) (**Figure 3** and **Supplementary Material S1**). However, tetracyclines, sulphonamides and nucleosides resistance genes were less abundant (**Figure 3** and **Supplementary Material S1**).

### Determination of Virulome Throughout Meat Chain Production

**FIGURE 4:**
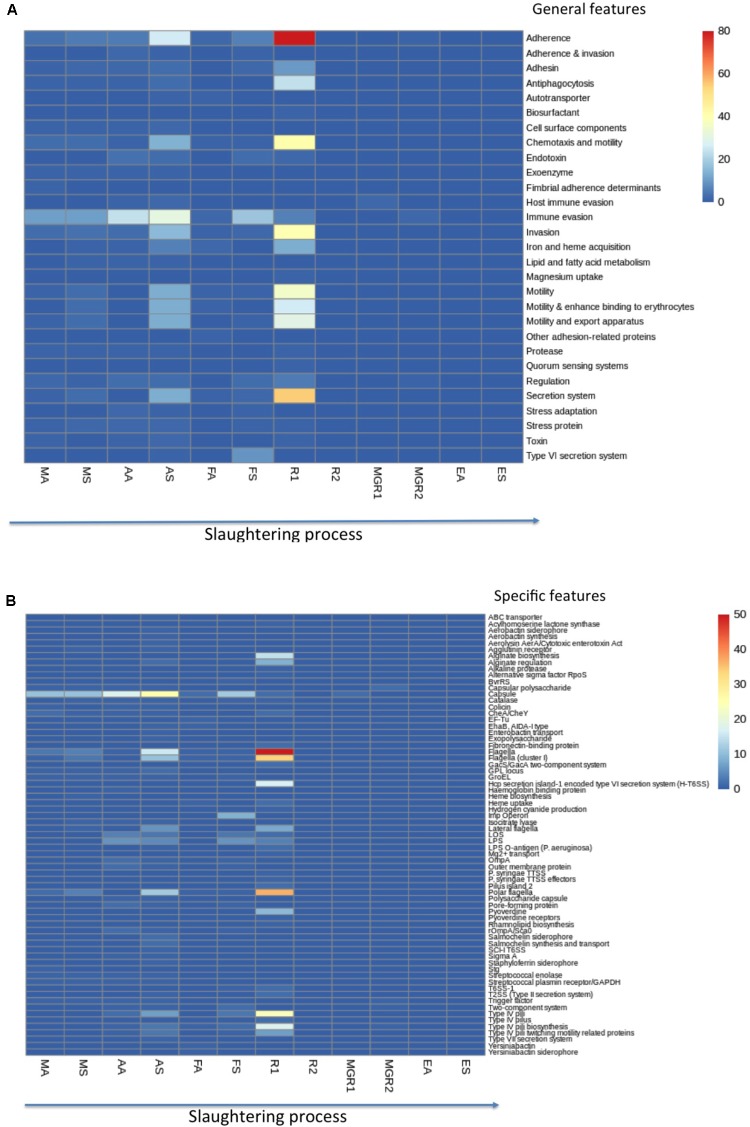
Heatmap showing the distribution of virulence genes detected within metagenomic samples of different slaughterhouse zone/product surfaces (MA, animal surface in Animal Arrival zone; MS, environmental surfaces in “animal arrival” zone; AA, animal surfaces in anesthesia zone; AS, process-area surfaces in anesthesia zone; FA, animal surfaces in “scorching and whip”; FS, process-area surfaces in “scorching and whip”; EA, animal surfaces in sale zone; ES, area surfaces in sale zone; R1 and R2, kidney; MGR1 and MGR2, lean). **(A)** Distribution of general VRGs throughout meat chain production; **(B)** distribution of specific VRGs throughout meat chain production. Colors shown in the legend (right) indicated the abundance of VRGs scaled from blue (undetected VRGs) to red (maximum; i.e., 80 VRG).

The incidence of virulence was assessed by metagenomic sequencing and compared with VFDB database. In regards to general classes of virulence determinants, R1 (kidney-surface sample) and AS (surfaces in the Anesthesia zone) exhibited more virulence traits than other zones/products, with up to 379 determinants in R1 and 174 determinants in AS (surfaces in anesthesia zone), followed by AA (animals in anesthesia) with 56 determinants, 52 determinants in FS (surface in “scorching and whip” zone), 46 determinants in MS (surfaces in “animal arrival”) and 35 in MA (“animal arrival”) (**Figure 4A** and **Supplementary Material S2**). However, MGR1 and MGR2 surface products and FA (animal surfaces in “scorching and whip”) had only 2, 5, and 13 virulence determinants, respectively; while R2 sample and those from sale zone (EA and ES) were free of virulence factors (**Figure 4A** and **Supplementary Material S2**). Among general virulence features: adherence, secretion system, chemotaxis and motility, invasion, motility and immune evasion were the most abundant (ranging from 32 to 81 determinants), especially in R1 sample followed by AS (**Figure 4A** and **Supplementary Material S2**); however, an immunity-evasion determinant was detected in almost every simple and most prevalent in AS. Other virulence factors detected included motility and export apparatus, motility and enhanced binding to erythrocytes, antiphagocytosis, iron and heme acquisition and Type IV secretion system (**Figure 4A** and **Supplementary Material S2**).

Further examination of specific virulence features revealed that flagella, polar flagella, flagella (cluster I), Type IV pili, capsule, Type IV pili, Type IV pili biosynthesis, Hcp secretion island-1 encoded type VI secretion system (H-T6SS), alginate biosynthesis and capsule were the most abundant (15–50 determinants) especially in R1 sample followed by AS (**Figure 4B** and **Supplementary Material S2**); however, the capsule virulence determinant was detected in almost every sample and most prevalent in AS. Other virulence features ranging from 5–10 determinant detections included alginate regulation, pyoverdine, lateral flagella, Type IV pili twitching motility related proteins, LPS and Imp operon (**Figure 4B** and **Supplementary Material S2**).

## Discussion

Animals are common reservoirs of several bacteria harbored by their skin, hair/wool, noses, urogenital and oral cavities, and also intestinal tract that could spread from the animals to the environment, and also from slaughterhouse to the food chain (Sobsey et al., 2006). The metaphylactic and prophylactic use (including use for growth promotion, which now has been banned in Europe since 2006) in agriculture and aquaculture has enhanced zoonotic bacteria as a major reservoir of antibiotic resistance (Food and Agriculture Organization of the United Nations [FAO], 2015, 2016). During slaughtering, animal hide, equipment, water, utensils and staff have been recognized as major sources of contamination (Bolton et al., 2002). Thus, contamination from the slaughtering and the processing of the carcasses could occur, spreading from one carcass to another, and could constitute a risk food-borne pathogen risk in humans. This is facilitated by the bacteria’s adaptability to multiple hosts, virulence mechanisms and high-rate of genetic exchange of antimicrobial resistance (Price et al., 2012; Lavilla Lerma et al., 2014b; Food and Agriculture Organization of the United Nations [FAO], 2015; Liu et al., 2016). These resistant bacteria, considered as superbugs, have become a global challenge and could result in pandemic situations with health and socio-economics repercussions (Padungtod et al., 2008; Fair and Tor, 2014; Founou et al., 2016), with food trade being a contributing factor.

In this study, we evaluated bacterial contamination levels in a porcine slaughterhouse throughout its production chain using culture-dependent and metagenomic methods. In terms of cultures, high loads of mesophilic bacteria (including pseudomonads, staphylococci, *E. coli* and LAB) were detected in all unit processes on both animal (and products) and process surfaces, especially in zones of: “animal arrivals” (MA and MS), stabling and corral showers (CA and CS), anesthesia (AA and AS), “scalding and depilation” (PA and PS), and “scorching and whip” (FA and FS) with viable counts ranging from 3.5–7.71 log_10_ CFU/ml. Similarly, Di Ciccio et al. (2016) found an average up to 6 log_10_ CFU/cm^2^ total viable counts in two pig-slaughterhouse plants in Northern Italy, which was equivalent to the counts obtained in some areas in this study (almost 6 log_10_ CFU/cm^2^). Furthermore, anesthesia zone (AA and AS) exhibited the highest counts followed by “scorching and whip” zone (FA and FS). However, the “scalding and depilation” process (intermediate zone between “anesthesia” and “scorching and whip”) significantly improved microbial safety by decreasing microbial counts by 1–3 orders of magnitude (log_10_), in a similar manner described by Gill and Bryant (1993) for *E. coli* following singeing. These results indicate that the major contamination sources involved handling of livestock hide, carcass, and viscera and process contamination as described elsewhere (Avery et al., 2002; Alegre and Buncic, 2004; Nastasijevic et al., 2008). As for animal products following evisceration, such as kidney, heart, stomach, liver, lean and lard (and also their transporting boxes), they exhibited either similar bacterial counts as their previous slaughterhouse zone (Scorching and whip, FA and FS), or even slightly higher counts in the case of pseudomonads, which suggest accumulated contamination from two main sources: the slaughterhouse environment (e.g., animals, equipment, and staff) and the gastro-intestinal tract of the animals. Thus, these microorganisms can cross-contaminate other products by using the same transport boxes.

The pathogens commonly associated with pork include *Staphylococcus aureus*, *Listeria monocytogenes, Campylobacter* spp., *Salmonella* spp., and *Yersinia enterocolitica* (Korsak et al., 1998; Koutsoumanis and Sofos, 2004; Malakauskas et al., 2006; Mataragas et al., 2008). The results in this study demonstrated that *St. aureus, L. monocytogenes*, and *Salmonella* spp. were highly recoverable from animal surfaces, surfaces within the slaughterhouse, viscera and meat products. *L. monocytogenes* and *Salmonella* spp. were present on almost all surfaces; however, some products also exhibited the presence of both pathogens such as kidney, lean and lard (*Listeria* sp.); liver, stomach, lean and their transport boxes (e.g., *Salmonella* sp.). Similarly, Sasaki et al. (2013) also detected several pathogens in swine livers collected at an abattoir, such as *Campylobacter* spp., *Salmonella* spp., and *L. monocytogenes*.

The presence of high microbial loads, especially pathogens in animal products destined to human consumption, constitute a challenge and reinforce the importance of controlling zoonotic pathogens in meat and other animal products through a complete and continuous farm-to-fork examination. In this sense, the high viable counts of all microbial groups found in the areas for anesthesia (AA and AS) and “scorching and whip” (FA and FS) indicate the importance of hygienic controls before further processing (evisceration and cutting) to prevent the spread of microorganisms downstream into the end products. Among these hygienic controls, cleaning and disinfection of anesthesia surface and scorching and whip band used for several animals (almost 200 animals) should be carried out after a determined number of animals (maximum of 10–20 animals) to reduce the cross-contamination and the increase of bacterial load in these surfaces. Cleaning procedures should be done with hot water under pressure, and disinfection protocols should be based on a rotatory use of several biocides not containing quaternary ammonium compounds and thus avoiding the emergence of resistance to the same biocide, for example the use of HLE disinfectant could be a good strategy (Abriouel et al., 2018).

Taking into consideration the results from the culture-dependent methods, further examination of antibiotic resistance genes and virulence determinants were conducted in selected slaughterhouse zones and products. Metagenomic analyses revealed a high diversity and richness of ARGs, up to 55–62 determinants in anesthesia area (AA and AS) and 35–40 in “animal-arrival zone” (MA and MS). However, “scorching and whip’ (FA and FS) area exhibited lowered abundance of ARGs (1–6), indicating that the scalding and depilating process (intermediate zone between “anesthesia” and “scorching and whip”), aimed to remove dirt and hair, had a significant effect in decreasing the resistome, as well as the microbiota (1–3 orders of magnitude). Although, an increase in microbial counts occurred in the subsequent “scorching and whip” zone (FA and FS). Thus, resistant bacteria gradually declined after scalding, leading to disappearance of these ARGs within the pan-microbial community, and thus we observed a decrease in ARG richness and diversity in meat and viscera products, and also in the slaughterhouse environment.

The high prevalence of antibiotic-inactivating enzyme genes (involved in the resistance to several antibiotics: mainly aminoglycosides, beta-lactams, lincosamides, macrolides, phenicols, tetracyclines, nucleoside antibiotics and streptogramin) in “animal-arrival zone” (60% in MA and 65% in MS) and anesthesia area (56% in AA and 56% in AS) were represented by the predominance of aminoglycoside (46–51%) and lincosamide (14–19%) resistance genes, which do not reflect the selective pressures exerted by tetracyclines and beta-lactams commonly used in porcine industry. The presence of mobile genes coding for aminoglycoside inactivation enzymes responsible for aminoglycoside resistance [*N*-acetyltransferases (AACs), *O*-nucleotidyltransferases (ANTs), and *O*-phosphotransferases (APHs)] in these zones represent a great challenge because of the spread of these genes to humans via meat products. In this sense, aminoglycosides are ‘critically important antibiotics’ such as amikacin, neomycin and kanamycin used for the treatment of severe systemic infections and also as a second-line antibiotic in combating multidrug-resistant tuberculosis (WHO, 2012); these antibiotics become inactivated by several enzymes encoded by resistomes in this study such as APH(3′)-IIIa and the broad-spectrum bifunctional enzyme AAC(6′)-Ie-APH(2″)-Ia, which are considered the most abundant aminoglycoside phosphotransferases in clinical settings and slaughterhouses (Tremblay et al., 2012; Woegerbauer et al., 2014). Furthermore, AAC(6′)-I is also the most prevalent and clinically relevant antibiotic inactivating enzyme present in most human Gram-negative pathogens (Ramirez et al., 2013) and also detected in several slaughterhouse zones in the present study. On the other hand, the kanamycin nucleotidyltransferase Ant(4′)-Ib, which confers resistance to kanamycin, neomycin and other aminoglycoside, was highly detected in slaughterhouse zones; it was also reported in meticillin-resistant *S. aureus* of human and animal origin (Otarigho and Falade, 2018). Regarding lincosamide resistance, lnuA, lnuB, and lnuC (coding for lincomycin resistance), detected in all studied slaughterhouse zones, were also detected in fecal microbiome of swine, farm workers and the surrounding villagers in China (Sun et al., 2017), and also in *S. aureus* isolates from healthy animals and sick populations in China (Liu et al., 2018).

Thus, pleuromutilins, polymyxins, aminoglycosides and lincosamides, used to a lesser extent, may have exerted sufficient selective pressures to maintain ARGs. Conversely, tetracyclines and beta-lactams most commonly administered to pigs appeared to have had minimal impact of selecting and maintaining their related resistance genes; only 1–4 tetracycline resistance genes were observed among 35-62 ARGs, such as *tet44, tetB, tetQ*, and *tetX* found in “animal-arrival zone” (MA and MS) and anesthesia area (AA and AS). Similar results were obtained by Looft et al. (2012) in swine intestinal microbiome of the antibiotic-fed pigs. This apparent paradox may be explained by the fact that several antibiotics were used in animal therapy (7–9 antibiotics per trimester), resulting in a diversification of resistance genes rather than a selective pressure by a single antibiotic. Or, alternatively, resistance to antibiotic pressures is being driven by generic efflux-pump mechanisms, rather than specific resistance genes.

Concerning virulence determinants, shotgun microbiome sequencing of VRGs evaluated their potential risk throughout meat production chain. In this sense, R1 sample obtained after evisceration constituted the main source of virulence determinants, being a conduit of more than 33 specific factors, represented by 19 classes of virulence features mainly adherence, secretion system, chemotaxis and motility, invasion and motility. However, an immunity-evasion (capsule and lipopolysaccharide) determinant was detected in almost every sample analyzed since the beginning of the slaughter process, with highest abundance in AS. Overall, virulence determinants detected in the pig slaughterhouse were related with pathogenic bacteria such as *Acinetobacter baumannii*, *Streptococcus* sp., *Pseudomonas* sp., *E. coli*, and *L. monocytogenes*.

In this sense, genes coding for flagella (flagella, polar flagella and lateral flagella; cluster I), capsule, Hcp secretion (island 1, T6SS), Type IV pili (island 2), lipopolysaccharide (O-antigen), pyoverdine and streptococcal enzymes (enolase, plasmin receptor/GAPDH) were harbored in slaughterhouse resistomes (environmental and animal surfaces, and also in end products). Type IV pili are the components of the PAPI-1 (pathogenicity island) conjugation machinery of *P. aeruginosa*, which is essential for the horizontal transmission of PAPI-1 (Carter et al., 2010). Furthermore, other island such as Hcp secretion (island 1), which encodes Type VI secretion system T6SS, was also found in slaughterhouse zones and is widely distributed in the human-pathogen *P. aeruginosa* and *Campylobacter jejuni* from poultry slaughterhouse (Ugarte-Ruiz et al., 2013). Specific virulence determinants of human pathogens (e.g., *P. aeruginosa* and *Streptococcus*, which can also affect animals), such as pyoverdine and streptococcal enzymes (enolase, plasmin receptor/GAPDH) were distributed in slaughterhouse zones.

We, as such, conclude that there are two main sources of contamination in the pig slaughterhouse: the first one is related with microorganisms of the animal hide, and the second one is related with evisceration step. Conversely to ARGs, VRGs were highly detected after evisceration in some products such as kidney, which reflect poor hygienic practices. Thus, this may be considered a threat to food safety and consumer health since bacteria with virulence determinants could cross-contaminate other products by via transporting boxes and processing surfaces. Slaughterhouses and meat processing represent a nexus of the “One Health” (or “One Medicine”) initiative that links human, animal and environmental health; as such control of ARG and VRG spread by adequate disinfection procedures in slaughterhouse becomes necessary to protect the health of human and animals.

## Conclusion

With high microbial loadings, and the high diversity and richness of ARGs and VRGs, control measures are required to reduce the risk of spread of pathogenic bacteria and their associated ARGs/VRGs in the slaughterhouse and meat products. In the pig slaughterhouse, there were two main sources of contamination: bacterial communities on the surface of the animals, and other related to evisceration. Thus, controlling these areas by adequate disinfection procedures may reduce risks to food safety and consumer health.

## Author Contributions

HA, NB, CK, and AG drafted the manuscript. HA designed the experiments. HA, NB, GCC, NCG, and BPM analyzed the data. All authors discussed the results, commented on the manuscript, and approved the final version.

## Conflict of Interest Statement

The authors declare that the research was conducted in the absence of any commercial or financial relationships that could be construed as a potential conflict of interest.

## Supplementary Material

**MATERIALS S1 and S2 |** Antibiotic resistance genes (ARGs) and virulence genes (VRGs) in a pig slaughterhouse, respectively.
